# Wnt/β-Catenin-Dependent Transcription in Autism Spectrum Disorders

**DOI:** 10.3389/fnmol.2021.764756

**Published:** 2021-11-11

**Authors:** Mario O. Caracci, Miguel E. Avila, Francisca A. Espinoza-Cavieres, Héctor R. López, Giorgia D. Ugarte, Giancarlo V. De Ferrari

**Affiliations:** ^1^Faculty of Medicine, Institute of Biomedical Sciences, Universidad Andres Bello, Santiago, Chile; ^2^Faculty of Life Sciences, Institute of Biomedical Sciences, Universidad Andres Bello, Santiago, Chile; ^3^Faculty of Veterinary Medicine and Agronomy, Nucleus of Applied Research in Veterinary and Agronomic Sciences (NIAVA), Institute of Natural Sciences, Universidad de Las Américas, Santiago, Chile

**Keywords:** Wnt/β-catenin signaling, transcription, chromatin remodeling proteins, synaptic dysfunction, autism spectrum disorders (ASD)

## Abstract

Autism spectrum disorders (ASD) is a heterogeneous group of neurodevelopmental disorders characterized by synaptic dysfunction and defects in dendritic spine morphology. In the past decade, an extensive list of genes associated with ASD has been identified by genome-wide sequencing initiatives. Several of these genes functionally converge in the regulation of the Wnt/β-catenin signaling pathway, a conserved cascade essential for stem cell pluripotency and cell fate decisions during development. Here, we review current information regarding the transcriptional program of Wnt/β-catenin signaling in ASD. First, we discuss that Wnt/β-catenin gain and loss of function studies recapitulate brain developmental abnormalities associated with ASD. Second, transcriptomic approaches using patient-derived induced pluripotent stem cells (iPSC) cells, featuring mutations in high confidence ASD genes, reveal a significant dysregulation in the expression of Wnt signaling components. Finally, we focus on the activity of chromatin-remodeling proteins and transcription factors considered high confidence ASD genes, including CHD8, ARID1B, ADNP, and TBR1, that regulate Wnt/β-catenin-dependent transcriptional activity in multiple cell types, including pyramidal neurons, interneurons and oligodendrocytes, cells which are becoming increasingly relevant in the study of ASD. We conclude that the level of Wnt/β-catenin signaling activation could explain the high phenotypical heterogeneity of ASD and be instrumental in the development of new diagnostics tools and therapies.

## Introduction

Autism (from the Greek root auto: self) was first described by Kanner (1943), who clinically characterized eleven cases, three girls and eight boys, with “extreme autistic aloneness” and “anxiously obsessive desire for the maintenance of sameness.” Since then, the definition of autism underwent constant revision to classify the pathology into a homogeneous subgroup. However, the wide arrange of disorders that shared common features lead to comprise these disabilities as autism spectrum disorders (ASD), thus widening the array of symptoms inside the diagnosis (Lai et al., 2014). To this day, Kanner described core symptoms required for the diagnosis of ASD under the Diagnostic and Statistical Manual of Mental Disorders (DSM; American Psychiatry Association). As of 2013 the DSM-5 has refined the criteria for diagnoses of ASD into two categories: persistent impairment in reciprocal social communication and social interaction; and restricted, repetitive patterns of behavior (Ozonoff, 2012). Defining the diagnosis criteria for ASD will continue to be a challenge in years to come, especially given the rising incidence in the population. For instance, in 1992 the estimate was that 1 in 500 children had ASD in the US, however, today it is estimated that 1 in 54 (Maenner et al., 2020) children carry this condition.^1^ Additionally, studies in Asia, Europe and North America have determined an average prevalence of about 1% for the general population (Kim et al., 2011). There are several potential explanations for an increase in the observed prevalence of ASD including the inclusion of milder neurodevelopmental differences bordering on normality, better identification and screening methods, broader diagnostic criteria, and increased awareness among parents and clinicians (Rice et al., 2012; Graf et al., 2017).

The genetic etiology of autism was early recognized by Kanner, the moment he noted that autistic children came from highly intelligent families, though better at relating to concepts than to people (Kanner, 1943). To date, family and twin studies have established ASD as a highly inheritable disease with a 90% phenotypic concordance among monozygotic twins (Rosenberg et al., 2009), with mutations ranging from single nucleotide variants (SNVs), copy number variants (CNVs) and large-scale structural variants (SVs), such as deletions, duplications, or translocations (Abrahams and Geschwind, 2008; O’Roak et al., 2012b). Significant advancements in identifying molecular mechanisms involved in ASD have been made by studying common ASD comorbidities with Mendelian inheritance patterns such as Tuberous Sclerosis (TSC1/2), Rett syndrome (MECP2), Fragile X syndrome (FMR1), Phellan-McDermid (SHANK3), Angelman (UBE3A), and Cowden (PTEN) syndromes, but altogether these disorders do not account for more than 10% of ASD cases (Sztainberg and Zoghbi, 2016).

Genetics and genome wide association studies (GWAS) have identified over 900 genes associated with ASDs (see SFARI Gene at: https://sfari.org/resources/sfari-gene). Nevertheless, most of the variants identified have a weak effect (Anney et al., 2012), suggesting a greater contribution for *de novo* occurring SNVs and CNVs (O’Roak et al., 2012b; Krumm et al., 2014; Lai et al., 2014). Indeed, 9% of *de novo* SNVs in affected individuals are disruptive or frameshift mutations that generate non-conserved amino acid changes, premature stop codons or alternative splice sites (Sanders et al., 2012; Iossifov et al., 2014), ultimately affecting the function of the resulting protein. Similarly, *de novo* CNVs are significantly enriched in individuals affected with the disorder and it is estimated that at least 8% of cases carry pathogenic variants (Levy et al., 2011; Sanders et al., 2012). Overall, it is estimated that these deleterious *de novo* variants contribute to about 30% of all simplex and 45% of female diagnoses (Iossifov et al., 2014; Sanders et al., 2015). In this regard, exome sequencing studies in family trios have identified that these disruptive *de novo* mutations are part of an interconnected network containing chromatin remodeling, synaptic and Wnt/β-catenin signaling genes (O’Roak et al., 2012a, b; De Rubeis et al., 2014; Turner et al., 2017).

**FIGURE 1:**
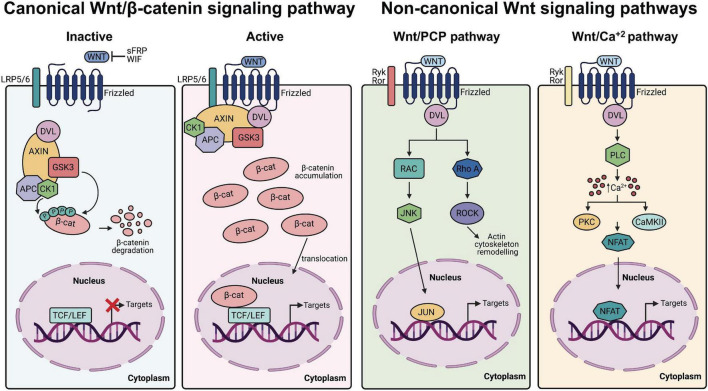
Canonical and non-canonical Wnt signaling pathways. From *left to right* Canonical Wnt/β-catenin. In the absence of canonical Wnt ligands (inactive state), the β-catenin destruction complex (AXIN, APC, DVL, CK1, and GSK3) allows the phosphorylation of β-catenin, which promotes its ubiquitination and proteasomal degradation, leading to repressed expression of Wnt/β-catenin target genes. Conversely, in presence of Wnt ligands (active state), the receptor complex composed of Frizzled and LRP5/6 recruits the destruction complex of β-catenin, which accumulates in the cytoplasm and translocate into the nucleus to promote Wnt/β-catenin target genes transcription. Non-canonical Wnt pathways. In the Wnt/PCP pathway, non-canonical Wnt ligands bind Ryk/Ror-Frizzled receptors and recruit Dvl leading to the activation of RhoA/ROCK and RAC/JNK. In the Wnt/Ca^2+^ pathway, the interaction of non-canonical Wnt ligands with Ryk/Ror-Frizzled increases the concentration of intracellular Ca^2+^ and activate downstream kinases and components of the signaling cascade.

Wnts are lipid-modified secreted glycoproteins that signal through at least three distinct signaling cascades: the canonical Wnt/β-catenin, non-canonical planar cell polarity and Wnt/Ca^2+^ pathways (De Ferrari and Inestrosa, 2000; Moon et al., 2004; Staal et al., 2008; Anastas and Moon, 2013; Nusse and Clevers, 2017; Figure 1). In particular, Wnt/β-catenin signaling is known to be essential for the development and function of the mammalian CNS where it participates in diverse biological processes, including neurogenesis and survival of neuronal hippocampal stem/progenitor cells (Lie et al., 2005; Kuwabara et al., 2009; Mardones et al., 2016), gliogenesis (White et al., 2010), formation and maintenance of pre and post-synaptic terminals (Ciani and Salinas, 2005; Speese and Budnik, 2007), axonal remodeling of cerebellar granule cells and branching of sensory axons in the spinal cord (Lucas and Salinas, 1997; Hall et al., 2000; Hollis and Zou, 2012) and excitatory and inhibitory synaptic transmission in the cerebellum, as well as in the hippocampal formation (Ciani and Salinas, 2005; Ahmad-Annuar et al., 2006; Chen et al., 2006; Beaumont et al., 2007; Avila et al., 2010; Cuitino et al., 2010).

Wnt/β-catenin signaling starts with the binding of the Wnt ligand to a 7-transmembrane helix Frizzled (FZD) receptor and members of the single pass transmembrane low-density lipoprotein (LDL) receptor related proteins (LRP5/6), which acts as co-receptors (MacDonald and He, 2012; Figure 1). Wnt binding triggers the dissociation of β-catenin from the destruction complex composed of Axin, adenomatous polyposis coli (APC) proteins and casein kinase 1 (CK1) and glycogen synthase kinase 3β (GSK3β) enzymes (Hart et al., 1998), resulting in cytosolic stabilization of β-catenin and its translocation to the nucleus where it interacts with transcription factors belonging to the T-cell factor/Lymphoid enhancing factor (TCF/LEF) family of proteins to activate transcription of Wnt/β-catenin target genes (Moon et al., 2004; Nusse and Clevers, 2017). Conversely, in the absence of Wnt ligands, Axin and APC proteins facilitate the sequential phosphorylation of β-catenin by CK1 and GSK3β tagging this protein for ubiquitination and proteasomal degradation (Stamos and Weis, 2013). Notably, several core components of Wnt/β-catenin signaling have been either genetically or functionally associated with ASD (De Ferrari and Moon, 2006; Martin et al., 2013; Caracci et al., 2016). For example, a rare missense variant of WNT1 (S88R) was found in lymphoblastoid cell lines from individuals with ASD and functional analysis of this variant using Wnt/β-catenin reporters in human embryonic kidney (HEK293T) tissue culture cells showed higher activation than wild-type WNT1 (Martin et al., 2013). LRP6 co-receptor deficiency in mice leads to decreased number of neurons in the deeper layers of the developing neocortex and develop a dramatically thinner cortical plate, cortical hypoplasia and neuroanatomical defects resembling those found in human epilepsy and intellectual disability (Zhou et al., 2004, 2006). Likewise, defects in the function of destruction complex components in forebrain pyramidal neurons such as APC (Mohn et al., 2014) and GSK3β (Mines et al., 2010; Latapy et al., 2012) or Axin in intermediate progenitors in the developing cerebral cortex (Fang et al., 2013, 2014), recapitulate ASD-like behaviors in model mice.

## Wnt/β-Catenin Signaling in Brain Development and Synaptic Function in Autism Spectrum Disorders

**FIGURE 2:**
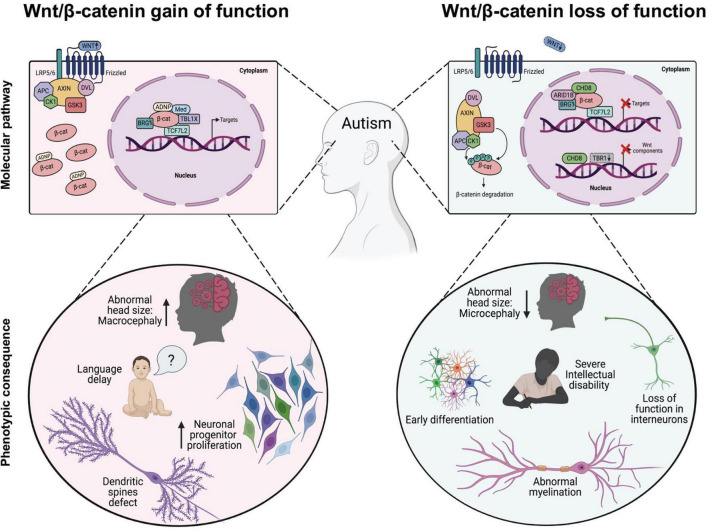
Dysregulation of Wnt/β-catenin-dependent transcription associated with phenotypic changes observed in autism spectrum disorders (ASD). In the Wnt/β-catenin-gain of function models (*left panel*), expression of transcription factors or chromatin remodeling proteins enhance Wnt/β-catenin signaling, either through direct binding with β-catenin that induces its cytosolic stabilization (i.e., ADNP) and/or its binding to target promoter region (i.e., MED12, TBL1X, BRG1, and TBL1XR1) leading to the appearance of some cellular and phenotypic traits observed in ASD. In the Wnt/β-catenin-loss of function models (right panel), inhibitory proteins interfere with the normal transcriptional activity of β-catenin through downregulating the expression of target genes (i.e., CHD8) or reducing the expression of functional components of the cascade, such as Fzd1, Fzd2, Dvl2, Dvl3, Wnt7b, and CTNNB1 (i.e., CHD8 and/or TRB1 cKo) thus promoting the appearance of other well studied cellular and phenotypic traits observed in ASD.

A common neuroanatomical feature observed in ASD children is a premature brain overgrowth occurring during the first 3 years of life, which affects the cerebral cortex, limbic system, hippocampal formation, amygdala, and cerebellum (Fombonne et al., 1999; Sparks et al., 2002; Courchesne et al., 2003; Figure 2). While the cortex is normally a laminar structure containing six layers with distinct molecular characteristics, an abnormal number and width of minicolumns has been associated with ASD (Amaral et al., 2008), including supernumerary neurons in layers I and the subplate and reduced neuron density in layers III, V, and VI (Hutsler and Casanova, 2016). Since a number of children with autism had more cortical neurons compared with control children, it has been proposed that the abnormally enlarged young ASD brains could be explained by excess number of neurons (Courchesne et al., 2011) and enhanced proliferation rates of neuronal progenitor cells (NPCs; Marchetto et al., 2017). Although the excess number of neurons is thought to underlie the excitatory/inhibitory balance of synaptic transmission that is critically affected in ASD (Yizhar et al., 2011; Nelson and Valakh, 2015), however, an excess cortical dendritic spines density has also been consistently observed in autistic patients and mice models (Zoghbi and Bear, 2012).

Early studies of Wnt ligands and their downstream signaling components in the mouse developing brain demonstrated that this cascade has an essential function in patterning the midbrain-hindbrain boundary, which later on gives rise to the brainstem and the cerebellum (McMahon and Bradley, 1990; Thomas and Capecchi, 1990; Hall et al., 2000), and forebrain derivatives such as the cerebral cortex, hippocampal formation and the amygdala (Grove et al., 1998; Lako et al., 1998; Houart et al., 2002; Maretto et al., 2003; Abu-Khalil et al., 2004; Zhou et al., 2004). Various Wnt/β-catenin ligands including Wnt1, Wnt3, Wnt3a, Wnt7a, and Wnt8a are involved in mammalian brain development and synaptogenesis (Braun et al., 2003; Caricasole et al., 2003; Ettenberg et al., 2010; Sharma et al., 2013; Wurst and Prakash, 2014). For instance, Wnt7a and Wnt8a have been shown to regulate excitatory synaptic formation and the LRP6 co-receptor is critical for the development of functional synapses *in vivo* (Ciani et al., 2011; Sharma et al., 2013). Moreover, a Wnt7a KO mouse shows reduced number of cortical progenitors due to increase differentiation of cells ultimately leading to microcephaly (Miao et al., 2018), and similarly, during cortical development the LRP6 KO mice display a smaller and thinner cortical plate with layer VI and layers II–IV showing a marked decreased in the number of neurons (Zhou et al., 2006), adding support that the Wnt/β-catenin complex receptor at the membrane has an essential role in cortical growth and lamination (Figure 2). Interestingly, mutations in proteins that promote the secretion of Wnt ligands such as PORCN (Castilla-Vallmanya et al., 2021) and Wntless (Chai et al., 2021) have also been recently described to regulate cortical development.

Downstream of the LRP6 co-receptor, the scaffold Disheveled (Dvl), which plays a central role in both canonical and non-canonical Wnt signaling (Gao and Chen, 2010; Gentzel and Schambony, 2017; Figure 1), was one of the first components of the signaling cascade to be functionally associated with various human psychiatric conditions, including ASD. Indeed, the Dvl1 KO mice exhibits sensorimotor abnormalities and abnormal social behavior, although their overall neuroanatomy seems intact compared to wild-type mice (Lijam et al., 1997). Next, Dvl1 was found to be important for synaptic vesicle clustering at pre-synaptic terminals and a novel Wnt7a/Dvl1 double mutant presented deficits in spine morphogenesis and excitatory neurotransmission (Ahmad-Annuar et al., 2006; Ciani et al., 2011), thus providing a molecular basis for the behavioral abnormalities observed in this model. Mammals share three Dvl family members with expression patterns and developmental functions partially redundant (Gentzel and Schambony, 2017). Interestingly, a compound KO of Dvl1^–/–^Dvl3^+/–^ in mice displayed autistic behaviors, without gross morphological brain abnormalities as described with the Dvl1 single KO, but with a transient increase in neuronal numbers in the deep layers of the neocortex at E14.5, which was later normalized at E18.5 days of development (Belinson et al., 2016). Surprisingly, abnormal development and post-natal autistic behaviors could be rescued by treatment with GSK3 inhibitors administered between E9.5 and E14.5, thus proving that Wnt signaling has differential roles during distinct developmental time points which ultimately shape neocortical development.

While Dvl deficiency may point out to a loss of function in Wnt/β-catenin signaling in ASD, an APC conditional KO (cKO) mouse in excitatory pyramidal neurons showed increased β-catenin levels and target Wnt/β-catenin gene transcription, accompanied at the cellular level, with an increase in dendritic spine density in the cortex/hippocampus and spines with a stubby phenotype indicative of immature synapses; and at the behavioral level, by learning impairments, reduced social interest and repetitive behaviors (Mohn et al., 2014). Conversely, it was found that a cKO of APC in dorsal cortical progenitors at E13.5 reduced the number of radial progenitors, leading to abnormal migration and differentiation of cortical precursors in a β-catenin/TCF4 dependent manner (Nakagawa et al., 2017).

Further involvement of the β-catenin destruction complex comes from studies of Axin, which is the main cytosolic scaffold for core Wnt/β-catenin signaling components, such as Dvl, APC, CK1, GSK3, and β-catenin (Behrens et al., 1998; Hart et al., 1998; Ikeda et al., 1998; Kishida et al., 1999; Figure 1). Axin is a critical regulator that determines the intermediate progenitors population size and the number of neurons during neurogenesis in the developing mouse cerebral cortex, an effect that is mediated by the interaction between Axin and GSK3 in the cytoplasmic compartments of the progenitors (Fang et al., 2013). Axin can be pharmacologically modulated by the tankyrase inhibitor XAV939, which inhibits β-catenin-mediated transcription and stimulates β-catenin degradation (Huang et al., 2009) and *in utero* injection of XAV939 in the lateral ventricle at E14.5 leads to a transient increase in the number of intermediate progenitors, ultimately increasing the number of pyramidal neurons in the neocortex within layer 2/3, which was enough to trigger an enlarged neocortex and autistic behaviors in mice (Fang et al., 2014; Fang and Yuste, 2017). Importantly, as development proceeds, Axin becomes enriched in the nucleus to trigger neuronal differentiation via β-catenin activation (Fang et al., 2013).

More recently, increased expression of β-catenin exclusively in mouse forebrain excitatory neurons lead to decreased social interest and repetitive behaviors, effectively mimicking ASD symptoms (Alexander et al., 2020). Conversely, de *novo* loss-of-function mutations in the CTNNB1 gene in ASD probands relates with microcephaly and severe intellectual disability (de Ligt et al., 2012). Interestingly, a cKO mice of β-catenin in parvalbumin interneurons, a major contributor of inhibitory outputs in the brain that are decreased in the prefrontal cortex of ASD individuals (Hashemi et al., 2017), leads to an increase in autistic behaviors while enhancing spatial memory (Dong et al., 2016). At the synapse, β-catenin is found in pre- and post-synaptic compartments of cultured hippocampal pyramidal neurons, where its deficiency reduces synaptic vesicle clustering and dendritic spine maturation (Bamji et al., 2003; Okuda et al., 2007). Accordingly, KO of APC that leads to increased cytosolic levels of β-catenin in pyramidal neurons increased dendritic spine density and ASD-like behavior (Mohn et al., 2014). Notably, double cKO of APC and β-catenin rescued ASD behaviors in mice (Alexander et al., 2020), suggesting that increased β-catenin activity is directly responsible for the phenotype.

Finally, fine-tuning of β-catenin levels throughout development is determinant for proliferation and differentiation of NPCs giving rise to the layered cerebral cortex, hippocampus and other brain areas (Figure 2). Indeed, β-catenin and other members of the catenin family are directly involved with adherent junctions and synaptic adhesion contacts, which makes them important scaffold proteins during mammalian CNS development (Yu and Malenka, 2003; Valenta et al., 2012). Notably, gain of function studies in mice over expressing constitutively active β-catenin displayed significant macrocephaly, with enlarged hippocampi and expansion of the cerebral cortex (Chenn and Walsh, 2003; Woodhead et al., 2006; Harrison-Uy and Pleasure, 2012). Enhanced proliferation rates of NPCs directed by Wnt/β-catenin signaling is supported by the finding that expression of inhibitors of β-catenin signaling in cortical precursors *in vivo* such as dominant-negative TCF4 (Korinek et al., 1997) or ICAT (Tago et al., 2000) results in premature cell cycle exit and neuronal differentiation (Woodhead et al., 2006). In this regard, the direct regulation of NPCs proliferation by Wnt/β-catenin signaling activation could underlie the occurrence of *de novo* somatic mutations in this cells. For instance, human NPCs derived from ASD patients with macrocephaly show increased proliferation and enhanced DNA damage (Wang M. et al., 2020). Interestingly, double strand breaks have been found enriched in actively transcribed regions where several genes associated with ASD are localized. Altogether this data shows that core upstream regulatory proteins strictly control β-catenin stability and that deficiency in any of this elements leads to synaptic and developmental defects commonly associated with ASD.

## Dysregulation of Wnt/β-Catenin Dependent Transcription in Autism Spectrum Disorders

Initial genome-wide transcriptional microarray analyses of human fetal neural progenitor cells incubated in the presence of human recombinant Wnt1 showed increased expression of Wnt effectors and several neuronal developmental pathways directly associated to neurodegenerative and neurodevelopmental disorders including ASD (Wexler et al., 2011). Wnt/β-catenin dependent transcription has been shown to enhance the expression of genes associated with synaptic transmission and neuronal excitability in cultured thalamic neurons (Wisniewska et al., 2012). Similarly, we have found in cultured rat hippocampal neurons treated with purified mouse recombinant Wnt3a that the transcriptional activity of β-catenin increases the expression of genes associated with forebrain development and neural precursor cell function (Perez-Palma et al., 2016). Moreover, we later found in these hippocampal cells that the autism-associated Neuroligin 3 gene (NLGN3; Jamain et al., 2003) is a direct target of β-catenin/TCF mediated transcription (Medina et al., 2018). More recently, recombinant Wnt3a and lithium treatment in cultured hippocampal neurons resulted in increased expression of several genes associated with pre and post-synaptic function, including Rim1, piccolo and Erc2 in the presynaptic compartment, as well as postsynaptic scaffolding markers such as postsynaptic density protein-95 (PSD-95/Dlg4), Homer1 and Shank1 (Martinez et al., 2020). Thus, Wnt/β-catenin dependent transcription has an essential activity on neuronal function and synaptic plasticity mechanism in neurons, which seems to be severely affected in ASD and related conditions (Zoghbi and Bear, 2012; Guang et al., 2018).

Considering the low availability of ASD brain samples to examine differentially expressed genes during brain development, initial transcriptomic studies focused on ASD-derived peripheral tissues such as blood and lymphoblastoid cell lines (Hu et al., 2006, 2009; Nishimura et al., 2007; Gregg et al., 2008; Lintas et al., 2012; Ansel et al., 2016). These studies revealed that there were significant alterations in the expression of genes involved in nervous system development, cell communication and immune response. More recently, analysis of leukocytes from 1- to 4-year-old male toddlers with ASD compared with typical development from the general population showed an increased transcription of ASD risk genes, which converged in gene networks associated to RAS-ERK, PI3K-AKT, and Wnt/β-catenin signaling pathways (Gazestani et al., 2019).

Consistent differences between ASD and normal brain samples have suggested that a large proportion of the ASD transcriptome exhibits transcriptional and splicing dysregulation in genes involved in neuronal and glial dysfunction (Voineagu et al., 2011; Gupta et al., 2014; Gandal et al., 2018; Li X. et al., 2019). While the assessment of transcriptomic organization in ASD brains is still limited, great efforts have recently been made to develop high-throughput platforms to study transcriptomics in induced pluripotent stem cells (iPSC) or in NPCs (Lin et al., 2016; DeRosa et al., 2018; Griesi-Oliveira et al., 2020). In this regard, initial studies in ASD-derived NPCs showed a decreased β-catenin-dependent transcriptional activity, increased proliferation and decreased synaptogenesis (Marchetto et al., 2017). More recently, a high throughput screening of isogenic iPSC lines, modified through CRISPR-Cas9 to generate loss of function mutations in 28 high confidence ASD genes, showed that they could be grouped in low Wnt/β-catenin signaling activity and decreased neurogenesis (enhanced proliferation) or high Wnt/β-catenin signaling and enhanced neurogenesis of hPSC differentiated into Pre frontal cortex neurons (Cederquist et al., 2020). More strikingly, iPSC lines showing decreased Wnt signaling activity (e.g., CTNNB1) correlate with normal language development while iPSC lines associated with a higher Wnt response (e.g., GSK3B) showed language delay, indicating that the level of activation of Wnt/β-catenin signaling could directly impact the severity of ASD phenotypes.

Wnt/β-catenin transcriptional dysregulation has also been consistently documented in patient-derived iPSCs and animal models for syndromic ASD. Syndromic ASD are neurological conditions which feature the basic symptomatology of ASD and are associated with chromosomal abnormalities or mutations affecting a single gene (Sztainberg and Zoghbi, 2016). For instance, mutations in SHANK3, a postsynaptic scaffold in glutamatergic synapses, leads to Phelan-McDermid Syndrome (Bonaglia et al., 2006) and in patient-derived iPSC it was found a reduced expression of genes associated with embryonic development, protein translation and Wnt signaling (Breen et al., 2020). SHANK3 directly interacts with β-catenin and mutant mice shows increased nuclear β-catenin and reduced localization in synapses (Qin et al., 2018), suggesting that the postsynaptic scaffold recruits β-catenin toward adherent junctions at the synapse. Similarly, mutations in the E3 ubiquitin ligase UBE3A gene are responsible for the Angelman Syndrome (Khatri and Man, 2019) and *de novo* ASD linked missense variant T485A was shown to enhance Wnt/β-catenin signaling activation through stabilization of β-catenin in HEK293T cells (Yi et al., 2017). More recently, RNA-seq of cortex and hypothalamus from Ube3a deficient mice showed dysregulation of Wnt target genes (Lopez et al., 2019).

Other syndromic ASD conditions including Tuberous Sclerosis (TSC1/2; Wiznitzer, 2004; Mills et al., 2017), Fragile X syndrome (FMR1; Luo et al., 2010) and Rett Syndrome (MECP2; Hsu et al., 2020) have also shown abnormal activation of Wnt signaling mediated transcription. For instance: (i) TSC1 and TSC2 form a functional complex with components of the β-catenin degradation complex, including GSK3β and Axin, which negatively regulate β-catenin stability and its activation of Wnt target genes (Mak et al., 2003, 2005); (ii) the fragile X mental retardation 1 (FMR1) gene product (FMRP) negatively regulates Wnt/β-catenin co-receptors controlling trans-synaptic signaling during synaptogenesis (Friedman et al., 2013); and (iii) Wnt/β-catenin signaling increases MeCP2 protein stability (Kweon et al., 2016), which in turn enhances Wnt/β-catenin pathway activation (Miao et al., 2013). Taken together, these data highlights the importance of Wnt/β-catenin signaling activity in both idiopathic and syndromic ASD.

## High Confidence Autism Spectrum Disorder Genes Coding for Chromatin Remodeling Factors Modulate β-Catenin Dependent Transcriptional Activity

**FIGURE 3:**
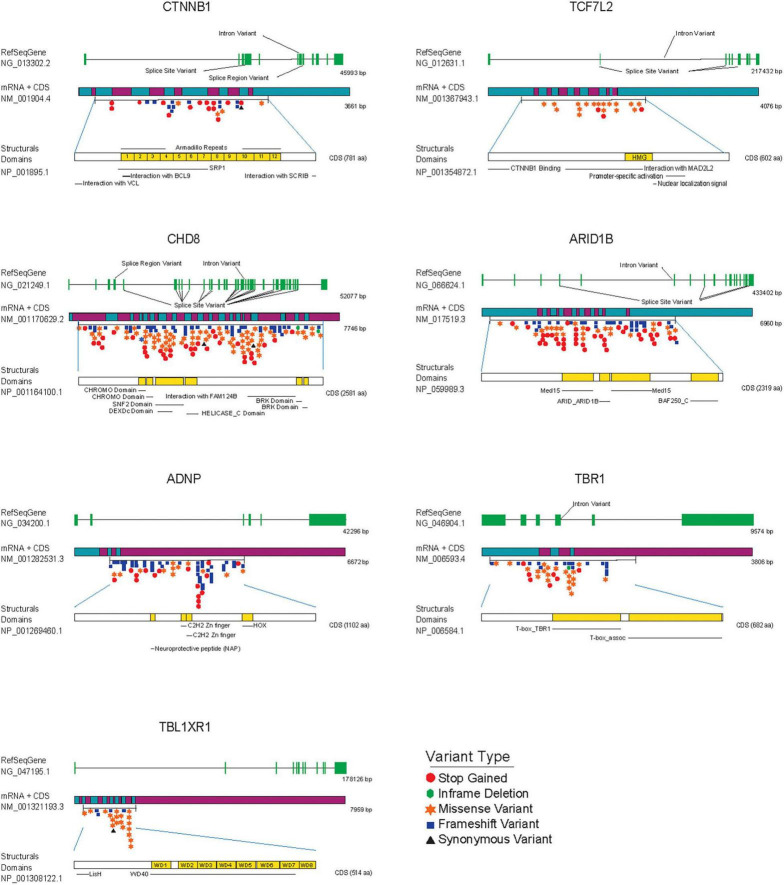
ASD *de novo* mutations in SFARI gene database associated with transcriptional components and chromatin remodeling factors modulating Wnt/β-catenin signaling. From *top to bottom* each figure shows: the reference gene used to identify mutations (RefSeqGene) with exons colored in green, the mRNA associated with the gene decorated with *de novo* mutations with the start and end of the coding sequence (CDS), and structural domains of each protein colored in yellow, as a reference to the importance of each mutation regarding structure and operation in general. Variants are highlighted and colored according to the Variant Type table.

The main downstream effectors of Wnt/β-catenin signaling in the nucleus are β-catenin and TCF/LEF transcription factors from the High Mobility Group (HMG) box family, which activate gene expression by recruiting other DNA-binding factors and chromatin remodeling proteins to promoter sequences of target genes (Cadigan and Waterman, 2012; Hrckulak et al., 2016; Table 1). Notably, the genes encoding for β-catenin (CTNNB1) and the TCF4/TCF7L2 transcription factor have been consistently associated with ASD and intellectual disability in several large-scale sequencing studies (de Ligt et al., 2012; O’Roak et al., 2012a, b; De Rubeis et al., 2014; Iossifov et al., 2014; Deciphering Developmental Disorders Study, 2015; Lelieveld et al., 2016; Marchetto et al., 2017; Satterstrom et al., 2020; Wang T. et al., 2020). Currently, CTNNB1 and TCF7L2 genes are considered high confidence genes for ASD in the SFARI Gene database,^2^ where there are 54 rare *de novo* variants reported in the CTNNB1 gene and 23 in the TCF7L2 gene (Figure 3), including missense, frameshift, stop gained and splice-site variants, clustered around functionally relevant protein-protein or protein-DNA interaction domains that remains to be characterized. In this regard, a recent characterization of 11 patients carrying *de novo* loss of function mutations in TCF7L2, within or near its HMG box domain, concluded that patients often show mild intellectual disability and neuropsychiatric conditions including ASD and ADHD (Dias et al., 2021). TCF7L2 is involved in neorcortical development and conditional inactivation of TCF7L2 in the forebrain leads to cortical hypoplasia, from reduced proliferation of intermediate progenitors and radial glial cells (Woodhead et al., 2006; Chodelkova et al., 2018; Bem et al., 2019). TCF7L2 deficient mice shows altered elongation of thalamocortical axons and decreased commitment to habenular and thalamic fates (Lee et al., 2017), resembling a previously documented phenotype in the LRP6 KO mice (Zhou et al., 2004). Finally, TCF7L2 has also been shown to regulate oligodendroglial development, oligodendrocyte differentiation (Hammond et al., 2015) and cholesterol biosynthesis during CNS myelinogenesis (Zhao et al., 2016), which may relate to abnormal myelination in the corpus callosum and decreased inter-hemispheric functional connectivity observed in individuals with ASD or other neurodevelopmental disorders (Anderson et al., 2011; Ameis and Catani, 2015; Li Q. et al., 2019).

**TABLE 1:** Wnt/β-catenin transcriptional regulators in autism spectrum disorders (ASD).

Gene	Model	Tissue/Cell type	*In vitro/vivo*	Wnt signaling	Evidence	References
CTNNB1	Mouse	Neuronal progenitor cells	*In vivo*	Expression of stabilized β-catenin. ΔN90β-catenin	Enlarged brain, increased neural precursor population	Chenn and Walsh, 2003
	Human	–	Patients	*De novo* loss of function mutation	Microcephaly and intellectual disability	de Ligt et al., 2012
	Mouse	PV+ interneurons	*In vivo*	cKO of Ctnnb1	Autistic behavior and enhanced spatial memory	Dong et al., 2016
	Mouse	APC/β-catenin cKO in forebrain excitatory neurons	*In vivo*	APC/β-catenin cKO	Single APC cKO leads to excess β-catenin and reduces social interaction. Double cKO of APC and β-catenin rescues social interaction defect	Alexander et al., 2020
TCF7L2	Human	–	Patients	–	*De novo* loss of function mutations. ASD, ADHD and intellectual disability	Dias et al., 2021
	Mouse	Brain, cortical progenitors electroporation at E13.5	*In vivo*	Expression of DN-TCF4	Cell cycle exit of cortical progenitors	Woodhead et al., 2006
	Mouse	Forebrain E11.5	*In vivo*	cKO of Tcf7l2	Hypoplastic forebrain, reduced proliferation of radial glia and intermediate progenitors	Chodelkova et al., 2018
	Mouse	Oligodendroglial linage cKO P7	*In vivo*	cKO of Tcf7l2	Tcf7l2 is required for oligodendrocyte differentiation independent of Wnt/β-catenin signaling	Hammond et al., 2015
CHD8	Human	–	Patients	–	ID, ASD, enlarged brain, neonatal hypotonia, and seizures	O’Roak et al., 2012b; De Rubeis et al., 2014; Iossifov et al., 2014
	Mouse	shRNA Chd8 *in utero* electroportation at E13 cortical progenitors	*In vivo*	Downregulation of Wnt pathway genes in neuronal linage cells	Reduced neural progenitor proliferation	Durak et al., 2016
	Human	Cell lines. HeLa, HCT116	*In vitro*	Direct binding to β-catenin and recruitment to β-catenin responsive promoters	Increased Wnt pathway gene expression in non-neuronal cells	Thompson et al., 2008
	Human	*CHD8*+/ – cerebral organoids	*In vitro*	Increased expression of Wnt signaling genes and TCF4	Transcriptomic analysis found significant overlap with idiopathic ASD brain organoids and iPSC neurons from BD patients	Wang et al., 2015
	Mouse	*Chd8 +/ –* in Olygodendrocyte precursor cells	*In vivo*	–	Decrease myelination and ASD behaviors	Kawamura et al., 2020
ARID1B	Human	–	Patients	–	ASD, ID, corpus callosum Agenesis	Halgren et al., 2012; Hoyer et al., 2012
	Mouse	Pyramidal neurons. In utero electroporation of shRNA against ARID1B in E14.5 embryos	*In vivo*	–	Decreased dendrite development. Aberrant dendritic spines and synaptic transmission	Ka et al., 2016
	Mouse	Arid1b +/ –	*In vivo*	–	Decreased number of GABAergic interneurons. Excitatory-inhibitory imbalance. Abnormal Social behaviors	Jung et al., 2017
	Human	Peripheral Lymphoblast of ARIDB1 loss of function mutations.	Patients	Upregulation of Wnt Pathway genes	RNA-seq	Vasileiou et al., 2015
	Human	HEK293T	*In vitro*	Defective binding of ID mutant ARID1B with β-catenin reduces Wnt target gene expression	Co-immnoprecipitation, Luciferace reporter essay	Vasileiou et al., 2015
	Zebrafish	*arid1b* knockdown	*In vivo*	Wnt target gene reduced expresión.	Growth delay as previously described for patients	Liu et al., 2020
ADNP	Human	–	Patients	–	*De novo* heterozygous mutations. Facial dysmorphism, speech impairment, motor dysfunction, intellectual disability	Helsmoortel et al., 2014
	Mouse	Adnp^+/^^–^	*In vivo*	—	Developmental delay, decreased vocalization, motor dysfunction, social impairment, dendritic spine defects	Hacohen-Kleiman et al., 2018
	Human	HEK293T		Binds to β-catenin and prevents association with Axin and APC	Co-immnoprecipitation	Sun et al., 2020
	Mouse	Embrionic Stem Cells Adnp ^–^/^–^	*In vitro*	Decreased Wnt-target gene expression	Decreased neuroectodermal genes expression	Sun et al., 2020
	Zebrafish	adnp –/–	*In vivo*	Decreased Wnt-target gene expression. Decreased β-catenin levels	Defective neuronal development	Sun et al., 2020
TBL1XR1	Human	HEK293T cell line transfected with TBL1XR1 Phe10Leu	*In vitro*	Increased TOP-Flash reporter and increased binding to β-catenin	Non-synonymus mutation found in Japanese ASD family trios.	Nishi et al., 2017
	Human	Cancer cell lines	*In vitro*	Direct binding of TBL1X and TBL1XR1 to β-catenin and recruitment to Wnt target promoters	Depletion of both proteins reduced Wnt signaling activation and oncogenic growth *in vitro* and *in vivo*.	Li and Wang, 2008
TBR1	Human	–	Patients	*De novo* loss of function mutations	ASD	Sanders et al., 2015
	Mouse	Tbr1^+/–^, Tbr1^layer 5^ cKO, Tbr1^Layer 6^ cKO	*In vivo*	Decreased Wnt7b expression, rescue of dendritic spine and social interaction defects using GSK3 inhibitors.	Decreased axonal thalamic arborization, dendritic spine development and social interaction defects	Fazel Darbandi et al., 2018

Recurrent mutations in the chromodomain helicase DNA binding protein 8 (CHD8) gene have been consistently identified in multiple individuals with ASD (O’Roak et al., 2012b; De Rubeis et al., 2014; Iossifov et al., 2014). Today, more than 200 of these rare variants are clustered throughout the gene, in coding regions for the helicase domain and other protein-protein interaction domains (Figure 3). CHD8 plays an important role in the transcription of genes related with the cell cycle and chromatin dynamics (Barnard et al., 2015) and knock down of this protein in NPCs disrupts expression of genes related to neuronal development and differentially alters the expression of multiple ASD-associated risk genes (Wilkinson et al., 2015; Durak et al., 2016). Notably, CHD8 binds β-catenin in the promoter regions of several β-catenin-responsive genes and negatively regulates β-catenin-dependent transcription in developing cortical neurons in mouse (Thompson et al., 2008; Durak et al., 2016). Moreover, knockout of one copy of CHD8 in human iPSCs revealed that CHD8 hemizygosity (*CHD8* +/-) affects the expression of thousands of genes in neural progenitors and early differentiating neurons (Wang et al., 2015), which are enriched for functions of neural development, Wnt/β-catenin signaling, extracellular matrix, and skeletal system development. Finally, as mentioned earlier for TCF7L2, CHD8 has an essential role in the proliferation-differentiation balance of oligodendrocyte precursor cells (Marie et al., 2018) and regulates the expression of several myelination-associated genes in these cells (Kawamura et al., 2020). Altogether these results implicate the activity of transcription and chromatin-remodeling factors in oligodendrocyte development and myelination, as an important piece of ASD neurological defects, and a new venue for Wnt/β-catenin signaling research (Bem et al., 2019).

The AT-rich interactive domain-containing protein 1B (ARID1B) gene has also been consistently associated with ASD and syndromic and non-syndromic ID, and corpus callosum agenesis (Nord et al., 2011; Halgren et al., 2012; Hoyer et al., 2012; O’Roak et al., 2012a; Stessman et al., 2017) and is similarly considered a high confidence gene for ASD with over 100 rare single gene variants (Figure 3). ARID1B codes for a member of the BRG1-associated chromatin-remodeling complex that uses the energy derived from ATP-hydrolysis to disrupt the chromatin architecture of target promoters (Collins et al., 1999; Wang et al., 2004; Trotter and Archer, 2008). It has been reported that knock down of ARID1B in the developing mouse brain leads to reduced dendritic arborization in cortical pyramidal and hippocampal neurons, accompanied by a decrease in dendritic spines density (Ka et al., 2016) and that ARID1B haploinsufficiency in mice leads to ASD-like behaviors and decreased GABAergic interneurons proliferation (Jung et al., 2017). In line with this evidence, patients with mutations in this protein often have epileptic seizures (van der Sluijs et al., 2019) and significant growth delay (Liu et al., 2020). As it was previously discussed for CHD8 factor, ARID1B associates and repress β-catenin-mediated transcription. Indeed, loss of function mutations in ARID1B results in transcriptional up regulation of Wnt/β-catenin target genes that leads to neurite outgrowth through β-catenin function (Vasileiou et al., 2015). More recently, ARID1B KO in chondrocytes reduced proliferation and inhibited differentiation by decreasing the expression of several genes associated to hypertrophy, alongside reducing expression of Wnt/β-catenin target genes (Liu et al., 2020). Hence, the role of ARID1B in multiple developmental processes from terminal development of pyramidal neurons and interneurons, to the regulation of chondrocyte development and the convergence of these phenotypes with Wnt/β-catenin functional activity make this protein an interesting ASD candidate gene and adds further support for the involvement of Wnt signaling in ASD and related developmental disorders.

Several other products that modulate Wnt/β-catenin-dependent transcription have been genetically or functionally associated with ASD, including the activity dependent neuroprotective protein (ADNP; Helsmoortel et al., 2014), members of the transducin β-like family TBL1X and TBL1XR1 (Choi et al., 2011; O’Roak et al., 2012a), the mediator complex subunits 12 (MED12; Kim et al., 2006; Lahbib et al., 2019) and 13L (MED13L; Utami et al., 2014), and the T-Box transcription Factor 1 (TBR1; Sanders et al., 2015; Figure 3). For instance, heterozygous *de novo* variants in ADNP deficiency often show intellectual disability, facial dysmorphism, speech impediments, and motor dysfunctions (Helsmoortel et al., 2014; Vandeweyer et al., 2014; Arnett et al., 2018). Interestingly, *ADNP* haploinsufficiency in mice recapitulates several of these characteristics and features reduced dendritic spine density and altered synaptic gene expression (Hacohen-Kleiman et al., 2018). Although ADNP is a presumed transcription factor that interacts with chromatin regulators of the SWI/SNF remodeling complex (Vandeweyer et al., 2014), little is known about its function as a transcriptional regulator. In this context, a recent study has found that ADNP stabilizes β-catenin through binding to its armadillo domain, which prevents its association with key components of the degradation complex and enhances Wnt/β-catenin transcriptional activation (Sun et al., 2020). Similarly, TBL1X and TBL1XR1 activate Wnt/β-catenin dependent transcription through direct association with β-catenin in the promoter regions of target genes in cancer cell lines (Li and Wang, 2008). Likewise, *de novo* non synonymous TBL1XR1 mutation associated with ASD (Phe10Leu) increased both binding with β-catenin and transcriptional activation of Wnt signaling in HEK293T cells, supporting the notion that that TBL1XR1 potentiation of Wnt signaling could be involved in ASD (Nishi et al., 2017). Interestingly, deletion Xp22.3, where TBL1X and NGLN4 genes are positioned, has been associated with several neurodevelopmental phenotypes and is highly penetrant in females (Chocholska et al., 2006). In the same X chromosome, MED12, which is involved in hindbrain patterning (Wang et al., 2006; Hong and Dawid, 2011) physically and functionally interacts with β-catenin *in vitro* and *in vivo* and is recruited to Wnt/β-catenin target genes in a β-catenin dependent manner (Kim et al., 2006). MED13L has also been associated with ASD and ID and downregulation of this protein in neural progenitor cells increases transcription of Wnt/β-catenin target genes (Utami et al., 2014). Finally, the transcription factor TBR1, which is highly expressed in the developing frontal cortex and affects the terminal differentiation of post mitotic neurons within the deep layers of the cortex (Bedogni et al., 2010) is involved in the expression of either Wnt7b (Fazel Darbandi et al., 2018), a known activator of Wnt/β-catenin pathway (Arensman et al., 2014) and potential ASD candidate gene (Sanders et al., 2015) or GSK3β, a negative regulator β-catenin transcriptional activity (Fazel Darbandi et al., 2020). Altogether, post-natal ablation of TBR1 in both layer 5 and 6 are sufficient to induce ASD-like behaviors in mice and ectopic activation of Wnt signaling with Wnt7b or GSK3β inhibitors rescues synaptic defects directly impacting social behaviors. Thus, regulation of GSK3β function to rescue synaptic defects in ASD offers an interesting pharmacological target and has received considerable attention recently (Liu et al., 2011; Caracci et al., 2016; Martin et al., 2018; Medina et al., 2018; Tatavarty et al., 2020).

In summary, deficiency in chromatin remodeling proteins and transcription factors associated with ASD widely recapitulate phenotypes resembled by β-catenin gain and loss of function experiments and highlights the importance of Wnt/β-catenin dependent transcription in ASD and related neurodevelopmental disorders.

## Concluding Remarks

The role and regulatory mechanisms controlling the activation and inhibition of Wnt/β-catenin signaling have been extensively studied in human disease (Moon et al., 2004; Clevers and Nusse, 2012). While it is widely accepted that gain of function mutations in Wnt/β-catenin signaling components underlies the onset or development of several cancers (Logan and Nusse, 2004; Reya and Clevers, 2005; Klaus and Birchmeier, 2008; Anastas and Moon, 2013; Ugarte et al., 2015), it is also recognized that Wnt/β-catenin loss of function mediates the development of neurodegenerative diseases including Alzheimer’s disease, cerebral ischemia, Parkinson’s disease, Huntington’s disease, multiple sclerosis, and amyotrophic lateral sclerosis (De Ferrari and Inestrosa, 2000; De Ferrari et al., 2003, 2007; De Ferrari and Moon, 2006; Purro et al., 2014; Libro et al., 2016). Interestingly, expression of genes associated with Wnt signaling regulation in ASD brains are also are also disrupted in AD brains (Zeidan-Chulia et al., 2014), and similarly, there is an extensive overlap in candidate genes associated with both cancer and ASD (Crawley et al., 2016). Such convergence of gain and loss of function of Wnt/β-catenin signaling in ASD is further supported by the essential function of β-catenin in brain development/growth or the differentiation/proliferation path for neuronal progenitors, suggesting the broadest of spectrums of Wnt signaling activation may underlie ASD development. Finally, different levels of activation of Wnt/β-catenin are correlated with language delay severity giving for the first time a glimpse in the phenotypic impact of Wnt signaling/β-catenin activation in ASD.

In the present work we reviewed the activity of Wnt/β-catenin signaling components in brain development and synaptic function in ASD that unequivocally points toward a dysregulation of Wnt/β-catenin associated targets. We further explore this observation by confirming that several transcriptional factors and chromatin remodeling proteins deemed ASD high confidence genes are also direct modulators of β-catenin mediated transcription. While we are reluctant to deem the levels of Wnt/β-catenin activation a “gray area,” we do think that both gain and loss of function of this pathway could explain the high heterogeneity of ASD. Furthermore, studies considering both temporal and spatial resolution of gene expression will be instrumental to fully comprehend this new “Wnt spectrum.”

The data presented in this article also highlights the growing potential of different cell types in the neurobiology of ASD, in these contexts, cell type specific loss of function in interneurons and oligodendrocytes leads to ASD, giving a rising importance the Wnt pathway beyond the traditional excitatory pyramidal neuron and the development of the cerebral cortex.

## Author Contributions

MC wrote the first draft and contributed to the overall structure of the manuscripts. MA revised the first draft, gave early contributions regarding content and designed and figures. FE-C and HL designed the figures and revised the final version. GU edited and contributed to the final manuscript version. GD wrote the manuscript, supervised overall writing and edited the final version. All authors contributed to the article and approved the submitted version.

## Conflict of Interest

The authors declare that the research was conducted in the absence of any commercial or financial relationships that could be construed as a potential conflict of interest.

## Publisher’s Note

All claims expressed in this article are solely those of the authors and do not necessarily represent those of their affiliated organizations, or those of the publisher, the editors and the reviewers. Any product that may be evaluated in this article, or claim that may be made by its manufacturer, is not guaranteed or endorsed by the publisher.

## Footnotes

1
https://www.cdc.gov

2
https://gene.sfari.org/database/human-gene/
